# GNA14, GNA11, and GNAQ Mutations Are Frequent in Benign but Not Malignant Cutaneous Vascular Tumors

**DOI:** 10.3389/fgene.2021.663272

**Published:** 2021-04-30

**Authors:** Philipp Jansen, Hansgeorg Müller, Georg C. Lodde, Anne Zaremba, Inga Möller, Antje Sucker, Annette Paschen, Stefan Esser, Jörg Schaller, Matthias Gunzer, Fabian Standl, Sebastian Bauer, Dirk Schadendorf, Thomas Mentzel, Eva Hadaschik, Klaus G. Griewank

**Affiliations:** ^1^Department of Dermatology, University Hospital Essen, University Duisburg-Essen and German Cancer Consortium (DKTK), Essen, Germany; ^2^Dermatohistologie am Stachus, Munich, Germany; ^3^Dermatopathologie Duisburg, Duisburg, Germany; ^4^Institute for Experimental Immunology and Imaging, University Duisburg-Essen, Essen, Germany; ^5^Institute of Medical Informatics, Biometry and Epidemiology (IMIBE), University of Duisburg-Essen, Essen, Germany; ^6^Department of Medical Oncology, University Hospital Essen, University Duisburg-Essen and German Cancer Consortium (DKTK), Essen, Germany; ^7^Dermatopathologie Friedrichshafen, Friedrichshafen, Germany; ^8^Dermatopathologie bei Mainz, Nieder-Olm, Germany

**Keywords:** vascular tumor, next generating sequencing, dermatology, oncogene, oncology

## Abstract

Cutaneous vascular tumors consist of a heterogeneous group of benign proliferations, including a range of hemangiomas and vascular malformations, as well as heterogeneous groups of both borderline and malignant neoplasms such as Kaposi’s sarcoma and angiosarcomas. The genetics of these tumors have been assessed independently in smaller individual cohorts making comparisons difficult. In our study, we analyzed a representative cohort of benign vascular proliferations observed in a clinical routine setting as well as a selection of malignant vascular proliferations. Our cohort of 104 vascular proliferations including hemangiomas, malformations, angiosarcomas and Kaposi’s sarcoma were screened by targeted next-generation sequencing for activating genetic mutations known or assumed to be potentially relevant in vascular proliferations. An association analysis was performed for mutation status and clinico-pathological parameters. Frequent activating hotspot mutations in *GNA* genes, including *GNA14* Q205, *GNA11* and *GNAQ* Q209 were identified in 16 of 64 benign vascular tumors (25%). *GNA* gene mutations were particularly frequent (52%) in cherry (senile) hemangiomas (13 of 25). In angiosarcomas, activating *RAS* mutations (*HRAS* and *NRAS*) were identified in three samples (16%). No activating *GNA* or *RAS* gene mutations were identified in Kaposi’s sarcomas. Our study identifies *GNA14* Q205, *GNA11* and *GNAQ* Q209 mutations as being the most common and mutually exclusive mutations in benign hemangiomas. These mutations were not identified in malignant vascular tumors, which could be of potential diagnostic value in distinguishing these entities.

## Introduction

The heterogeneous group of cutaneous vascular tumors comprises both benign and malignant tumors (Llamas-Velasco and Mentzel, 2020). Cutaneous hemangiomas are benign and the most frequent tumors of vascular endothelial origin. Depending on the age, different subtypes of hemangioma predominantly occur that can regress completely. Clinical appearance of these hemangioma subtypes varies. Cherry hemangioma as one of the most common hemangiomas in adult life manifests as a sharply demarcated red nodule with a diameter of several millimeters. Most forms of hemangiomas are now grouped as variants of lobular hemangioma, including lobular capillary hemangioma (pyogenic granuloma). Additionally, various malformations can present which can involve arterial, venous or lymphatic vascular structures either alone or in combination (e.g., lymphovenous malformation). Some of these subtypes were previously, and to some extent are still, referred to as cutaneous cavernous hemangiomas (Mentzel et al., 1994; Elgoweini and Chetty, 2012; Qadeer et al., 2020). Thus, vascular lesions biopsied or excised for pathological evaluation can either represent newly acquired tumors or early or late manifestations of vascular malformations.

Cutaneous angiosarcomas are rare aggressive, malignant endothelial-cell tumor of lymphatic or vascular origin, representing less than 5% of all cutaneous sarcomas. They occur most frequently as sporadic tumors (primary angiosarcoma) on sun-damaged skin of the head and neck, and less commonly secondary to predisposing factors. Secondary angiosarcomas typically develop after radiation therapy for breast cancer (radiation-associated angiosarcoma) or in the setting of chronic lymph edema (Stewart Treves syndrome). Angiosarcomas have high rates of local recurrence after surgical excision and metastasis due to their intrinsic biologic properties and often delayed diagnosis, leading to a poor prognosis and a high mortality rate. The histopathological spectrum of cutaneous angiosarcoma ranges from morphologically low grade tumors showing well-formed vessels with mild nuclear and cytologic atypia, to morphologically high grade tumors displaying severe pleomorphism, mitotic activity and solid growth pattern resembling undifferentiated pleomorphic sarcoma (Brenn, 2014; Shon and Billings, 2017; Shustef et al., 2017).

Kaposi’s sarcoma is defined as an aggressive or borderline vascular tumor that is associated with the presence of human herpesvirus 8 (HHV 8) and shows pronounced growth under immunosuppression (Chang et al., 1994). Four principal clinical variants of Kaposi’s sarcoma are identified: classical, African-endemic, immunosuppressive drug-related (post-transplant) and acquired immunodeficiency syndrome (AIDS)-related. The classical type occurs predominantly in older men with a Mediterranean background. Kaposi’s sarcoma can occur in one anatomical region or disseminated over the whole body affecting both the skin and visceral organs (Hengge et al., 2002).

The tumors are conventionally distinguished by histopathological evaluation of morphologic characteristics and by immunohistochemical markers. Despite the recent wide-spread clinical application of next generation sequencing methodology, pathogenesis of vascular tumors remains incompletely understood. For cherry, congenital, anastomosing hemangioma, *GNA14, GNA11 and GNAQ* alterations have been described to be the most common alterations in these subtypes (Ayturk et al., 2016; Bean et al., 2017; Liau et al., 2019). For radiation-associated and lymphedema-associated angiosarcoma high-level amplification of the proto-oncogene *MYC* is regarded one of the most common genetic alterations (Guo et al., 2011; Mentzel et al., 2012; Shon et al., 2014; Harker et al., 2017; Requena et al., 2018). In a recent report, whole-exome sequencing of 47 angiosarcomas revealed a heterogenous mutational landscape with 30 genes recurrently mutated including *KDR*, *TP53* and *PIK3CA* (Painter et al., 2020). Angiosarcomas arising in sun-exposed regions demonstrated a UV-mutation profile with high tumor mutational burden. Hemangioendothelioma are rare vascular neoplasms classified as borderline (e.g., Kaposi’sform hemangioendothelioma) or malignant (e.g., epitheloid hemangioendothelioma) neoplasms. Some of these tumors are found to harbor highly specific, defining genetic alterations (e.g., the *WWTR1-CAMTA1* fusion in epithelioid hemangioendothelioma) (Flucke et al., 2014).

Assessing and comparing the genetic mechanisms occurring in different vascular tumors contributes to a better understanding of these tumors. This knowledge may potentially be helpful for classifying tumors where distinguishing dignity and tumor type solely by histomorphological assessment is challenging.

## Materials and Methods

### Sample Selection

Vascular tumor samples were obtained searching the databases of the Department of Dermatology University Hospital Essen (*n* = 38), Dermatopathologie bei Mainz (*n* = 43), Dermatopathologie Duisburg (*n* = 8) and Dermatopathologie Friedrichshafen (*n* = 15), Germany. All cases were screened by at least one experienced board-certified histopathologist (KG, EH, JS, TM, HM). The study was done in accordance with approval of the ethics committee of the University of Duisburg-Essen (IRB-number 20–9688-BO). The angiosarcoma cohort included 11 samples previously reported (Murali et al., 2015).

### Clinical and Histopathological Analysis

Available clinical information was taken from histology reports and patient records. Histological assessment included distinction between hemangiomas, malformations, angiosarcomas and Kaposi’s sarcoma. The subtype of hemangioma and malformations was taken from the pathology reports and reviewing available slides and immunohistochemistry. Kaposi’s sarcoma diagnosis were confirmed applying HHV8 immunohistochemical stainings. Cherry hemangioma are not included in the current classification of the society for Vascular Anomalies (ISSVA) for benign vascular tumors although they have distinct clinical and histopathological features. Due to clinical frequency of the use and comparability with the few former publication on genetic alterations in vascular tumors, we still classified hemangiomas as cherry hemangioma not listed as an independent entity according to ISSVA (2018).

### DNA Isolation

DNA was isolated from 10 μm-thick sections, cut from formalin-fixed, paraffin-embedded tumor tissues. The sections were deparaffinized and the whole tissue was manually macrodissected. DNA isolation was performed with the QIAamp DNA Mini Kit (Qiagen, Hilden, Germany) according to the manufacturer’s instructions.

### Targeted Sequencing

A custom amplicon-based sequencing panel covering 16 genes (Supplementary Table 1) was designed and prepared applying the GeneRead Library Prep Kit from QIAGEN^®^ according to the manufacturer’s instructions. Adapter ligation and barcoding of individual samples were done applying the NEBNext Ultra DNA Library Prep Mastermix Set and NEBNext Multiplex Oligos for Illumina from New England Biolabs. Up to 60 samples were sequenced in parallel on an Illumina MiSeq next generation sequencer. Sequencing analysis was performed applying the CLC Cancer Research Workbench from QIAGEN^®^. In brief, the following steps were applied. The workflow in CLC included adapter trimming and read pair merging before mapping to the human reference genome (hg19). Insertions and deletions as well as single nucleotide variant detection, local realignment and primer trimming followed. Additional information was then obtained regarding potential mutation type, known single nucleotide polymorphisms and conservation scores by cross-referencing varying databases (COSMIC, ClinVar, dbSNP, 1000 Genomes Project, HAPMAP and PhastCons-Conservation_scores_hg19). The resulting csv files were further analyzed manually. Mutations affecting the protein coding portion of the gene were considered if predicted to result in non-synonymous amino acid changes. The mean coverage of the targeted sequencing region achieved in all samples was 7479.5 reads. Mutations were reported if the overall coverage of the mutation site was ≥30 reads, ≥5 reads reported the mutated variant and the frequency of mutated reads was ≥2%.

## Results

### Sample Cohort

The study cohort consisted of 104 vascular tumor samples from 102 patients. We examined 64 hemangiomas and vascular malformations (28 females and 35 males) with an average age of 53 years (range 21–80), 19 angiosarcomas (15 females and 4 males) with an average age of 67 years (range 43–88), 21 Kaposi’s sarcomas (4 females and 16 males) with an average age of 56 years (range 28–84). In the cohort of angiosarcoma, previous postsurgical radiotherapy after carcinoma of the mamma was known to have been performed in 7 patients of the cohort. In 10 angiosarcomas with known copy number data, the 3 samples with MYC amplifications all had developed post radiotherapy. One HRAS Q61L mutation could be identified in an (secondary) angiosarcoma that had developed after radiotherapy due to breast cancer. The other HRAS Q61L mutation and the NRAS G12D were both identified in (primary) idiopathic angiosarcomas, respectively. In the cohort of Kaposi’s sarcoma (KS) 4 patients were known to have an HIV infection. All samples were primary tumors. Available clinical data are listed in Table 1.

**TABLE 1:** Associations of mutations with clinical variables for different vascular tumors and malformations.

		All	WT	*GNA14 mutant*	*GNA11 mutant*	*GNAQ mutant*	*NRAS mutant*	*P*-value*

Hemangioma/vascular malformation								

		*n* = 64	*n* = 46	*n* = 9	*n* = 2	*n* = 5	*n* = 2	
Mean age (years)		53	54	47	61	46	60	0.39
Sex	Female	28	23	2	1	2	0	0.46
	Male	35^#^	23	6^#^	1	3	2	
Sites of involvement	Head/neck	21	17	1	0	2	1	An error in the conversion from LaTeX to XML has occurred here. 6*0.29
	Ventral trunk	16	8	4	0	3	1	
	Dorsal trunk	13	7	4	2	0	0	
	Upper extremity	5	5	0	0	0	0	
	Lower extremity	7	7	0	0	0	0	
	LND	2	2	0	0	0	0	

		**All**	**WT**	***HRAS mutant***	***NRAS mutant***	*P*-value*		

**Angiosarcoma**								

		***n* = 19**	***n* = 16**	***n* = 2**	***n* = 1**			

Mean age (years)		67	65	75	74	0.56		
Sex	Female	15	12	2	1	1.0		
	Male	4	4	0	0			
Sites of involvement	Head/neck	8	6	1	1	1.0		
	Ventral trunk	7	6	1	0			
	Dorsal trunk	0	0	0	0			
	Upper extremity	0	0	0	0			
	Lower extremity	1	1	0	0			
	LND	3	3	0	0			

		**All/WT**						

**Kaposi’s sarcoma**								

		***n* = 21**						

Mean age (years)		56						
Sex	Female	4						
	Male	16^!^						
An error in the conversion from LaTeX to XML has occurred here. 6*Sites of involvement	Head/neck	2						
	Ventral trunk	2						
	Dorsal trunk	0						
	Upper extremity	2						
	Lower extremity	13						
	LND	2						

*LND, localization not determined; WT, wild-type for *GNA14, GNA11, GNAQ*, HRAS, KRAS, and *NRAS*. ^#^One male had two hemangiomas both showing a GNA14 mutation. ^!^One male had two Kaposi’s sarcoma. ^∗^Age – Kruskal-Wallis test; all others – Fisher exact test.*

### Mutation Analysis for Activating Oncogene Driver Mutations

**FIGURE 1:**
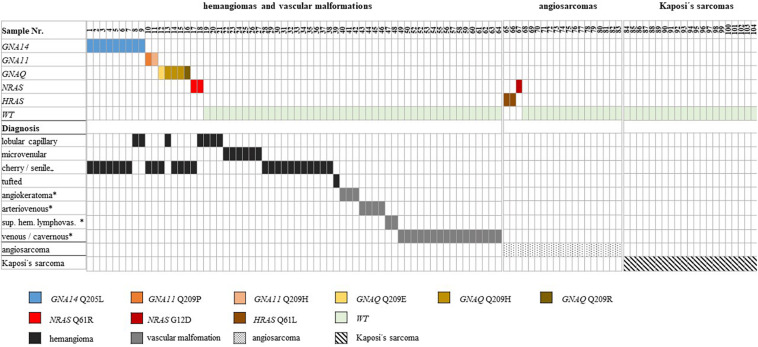
Distribution of activating mutations identified in vascular tumors. Distribution of activating mutations identified in different oncogenes in the vascular tumor cohort. The resulting amino acid changes are color-coded according to the scheme underneath the illustration [_+_ not listed as an independent entity according to ISSVA (2018), * classified as malformations according to ISSVA, sup. hem. lymphovas. = superficial hemosiderotic lymphovascular (previously referred to as targetoid hemosiderotic)].

**FIGURE 2:**
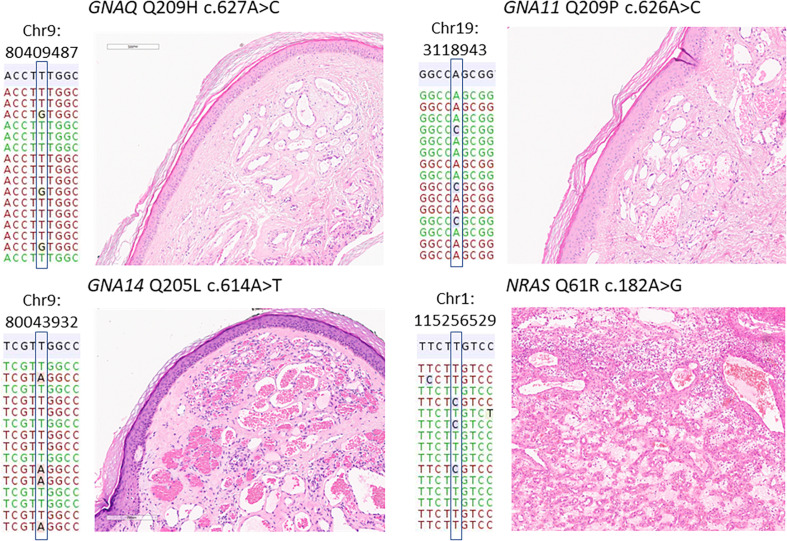
Representative mutations and histology in benign vascular proliferations. Representative histological images and the respective mutations identified in four vascular tumors. The mutations are annotated according the human genome build 19 (hg19). The reference hg19 sequence is shown in gray, below a representative selection of reads, some of which harboring the mutated nucleotide. All vascular tumors shown here were diagnosed as lobular hemangioma, the *NRAS* mutant case (bottom right) as the subtype eruptive hemangioma.

In the cohort of benign hemangiomas and vascular malformations, 18 mutations (Table 2) could be detected in 64 samples (28%). Targeted amplicon Next-Generation sequencing identified 9 GNA14 c.614A > T, Q205L mutations (Figures 1, 2). Five GNAQ mutations, consisting of 3 c.627A > C, Q209H, 1 c.626A > G, Q209R and 1 c.624_625delinsTG, Q209E alterations were observed. GNA11 Q209 mutation were identified in 2 case, comprising 1 c.626A > C and 1 c.627G > C alterations. NRAS mutations were identified in 2 cases being c.182A > G Q61R alterations. In the cohort of angiosarcomas, mutations were identified in 3 tumors. 2 HRAS c.182A > T, Q61L mutations and 1 NRAS c.35G > A, G12D mutation. In Kaposi’s sarcomas, no activating oncogene driver mutations could be detected. All mutations identified are known to be functionally activating and were found to be mutually exclusive (Figure 1).

**TABLE 2:** Mutations identified in vascular tumors and malformations.

Family	Gene	AA	cDNA	Hem/MF	AS	KS
*GNA*	*GNA14*	Q205L	c.614A > T	9		
	*GNA11*	Q209P	c.626A > C	1		
		Q209H	c.627G > C	1		
	*GNAQ*	Q209H	c.627A > C	3		
		Q209E	c.624_625delinsTG	1		
		Q209R	c.626A > G	1		
*RAS*	*HRAS*	Q61L	c.182A > T		2	
	*NRAS*	Q61R	c.182A > G	2		
		G12D	c.35G > A		1	

*AA, aminoacid alteration; Hem, hemangioma; MF, malformation; AS, angiosarcoma; KS, Kaposi’s sarcoma.*

### Associations of Clinical and Pathological Parameters With Oncogene Mutation Status

An analysis with available clinico-pathological data was performed. In the three different cohorts, statistically significant associations could not be detected between oncogene mutation status and patient age, sex and sites of involvement, respectively (Table 1). For hemangioma and vascular malformations, patients with GNA14 mutations (*n* = 9) and GNAQ (*n* = 5) tended to be younger than the average cohort. The frequency of GNA gene mutations (GNAQ, GNA11 and GNA14) was highest with more than 52% in cases diagnosed as cherry (or senile) hemangioma. Statistically significant associations between mutation status and histological subtypes could not be found in our cohort (Table 3). In the cohort of 19 angiosarcomas, the three mutant angiosarcomas arose in older patients than wild-type tumors. Occurrence of mutations in women was statistically non-significant for prior radiotherapy.

**TABLE 3:** Associations of mutations with histological subtypes of hemangiomas and vascular malformations.

	All	WT	*GNA14*mutant	*GNA11* mutant	*GNAQ* mutant	*NRAS* mutant	*P*-value*
	*n* = 64	*n* = 46	*n* = 9	*n* = 2	*n* = 5	*n* = 2	
Lobular capillary/pyogenic granuloma	7	3	2	0	1	1	
Microvenular	6	6	0	0	0	0	
Cherry/senile^#^	25	11	7	2	4	1	
Tufted	1	1	0	0	0	0	
Angiokeratoma^+^	3	3	0	0	0	0	0.17
Arteriovenous^+^	4	4	0	0	0	0	
Superficial hemosiderotic lymphovascular ^+*x*^	2	2	0	0	0	0	
Venous/cavernous^+^	16	16	0	0	0	0	

*^+^Classified as malformations according to ISSVA (2018). ^#^Not listed as an independent entity according to ISSVA. ^*x*^Previously referred to as targetoid hemosiderotic. **^∗^**Fisher exact test.*

## Discussion

We comparatively analyzed a cohort of vascular tumors comprising benign hemangiomas, vascular malformations, angiosarcomas and Kaposi’s sarcomas by targeted next generation sequencing. In line with two recently published cohorts of 85 hemangiomas, we also found *GNA14* and *GNAQ* alterations to be the most common mutations in our cohort of benign vascular tumors. The overall mutation frequency was considerably lower in our study, with 25% compared to the published 82% (Liau et al., 2019). However, the difference in the percentage value is certainly mainly due to our non-predefined histological inclusion criteria of subtypes whereas the described cohort included only cherry and cherry hemangioma like hemangiomas. The mutations were primarily found in hemangiomas and in particular those diagnosed as cherry (or senile) hemangioma. Here, the mutation frequency for *GNA* gene (*GNAQ, GNA11, GNA14*) mutations was highest, at 52%. Our findings demonstrate that *GNA* gene (*GNAQ, GNA11, GNA14*) mutations are a major pathogenetic event in benign vascular proliferations, however, primarily in hemangioma and not vascular malformations.

We did however, also identify 2 activating *NRAS* Q61R mutations in our cohort of benign vascular proliferations. Here similar to GNA gene mutations, *RAS* mutations were only identified in hemangiomas not in vascular malformations. *RAS* mutations have been previously described in vascular tumors including lobular capillary hemangiomas (pyogenic granuloma) (Lim et al., 2015; Murali et al., 2015; Groesser et al., 2016). Our findings demonstrate, that while much less frequent than *GNA* gene mutations, they do occur in hemangioma. As they were detected in a mutually exclusive fashion with *GNA* family gene mutations, it suggests only one of these mutations is required for vascular tumor formation to occur.

The distribution of *GNA* mutations we identified is interesting. As mentioned, activating *GNAQ* and *GNA11* mutations were first identified in cutaneous blue nevi and uveal melanoma (Van Raamsdonk et al., 2009, 2010). The mutations observed in cutaneous hemangioma alter the same conserved glutamine residue (Q209), however, as observed in other studies (Le Guin et al., 2019), the replacing amino acid in vascular tumors is different from melanocytic tumors. In melanocytic tumors, the recurring alterations are *GNAQ* Q209L or Q209P and *GNA11* Q209L. In hemangioma *GNAQ* mutations were found to lead to Q209H, Q209R and Q209E alterations, in *GNA11* the mutations resulted in Q209H and Q209P alterations. *GNA14* mutations have not been reported in melanocytic tumors, but were the most frequent mutations observed in our cohort of benign hemangioma and vascular malformations (*n* = 9). All mutations occurred at the identical conserved glutamine site resulting in Q205L. Different mutation profiles observed in *GNA* family members between melanocytic and vascular tumors, is likely linked to different functional consequences, a hypothesis that will however, need to be explored in future functional studies.

Mutations in GNA14, GNA11 and GNAQ were found to be mutually exclusive. These genes share ∼90% sequence homology and all belong to the GNAQ gene family. GNA15 is also a member of the GNAQ family, which is why we included the corresponding Q212 hotspot in our panel. However, no alterations were observed, suggesting that in contrast to the other GNAQ family members, mutations in GNA15 are not relevant in the pathogenesis of cutaneous vascular tumors.

In our cohort of angiosarcomas no alterations in *GNA14* or *GNA11* were identified. Fitting previous studies of angiosarcomas (Behjati et al., 2014; Murali et al., 2015; Painter et al., 2020), in 3 of 19 analyzed angiosarcomas (16%) activating *RAS* mutations [2 *HRAS* (Q61) and 1 *NRAS* (G12)] were identified. The mutational heterogeneity in angiosarcoma has also been demonstrated by previous studies and is at least in part explained by the different etiological factors of angiosarcomas including chronic sun exposure, radiation exposure and lymphostasis. Due to the limited number of angiosarcomas showing *RAS* mutations in our cohort, no clinical relevance of the shift in median age to wild-type angiosarcoma can be concluded. In the analyzed Kaposi’s sarcoma no activating gene mutations were identified. This fits with previous studies reporting few genetic or cytogenetic abnormalities aside from human herpes virus 8 (HHV8) (Sun et al., 2014). This data further supports HHV8 being the key modulator in the development of Kaposi’s sarcoma.

The distinction in mutation distribution was prominent. Hotspot mutations in *GNA* or *RAS* mutations were only observed in hemangioma, not in vascular malformations. Also, whereas rare *RAS* mutations were found in both benign hemangiomas and angiosarcomas, *GNA* gene mutations were only detected in benign hemangiomas. Distinguishing hemangiomas from vascular malformations and malignant angiosarcomas is generally straightforward with routine histopathology and the aid of immunohistochemistry, however, in some cases it can prove very challenging, especially if small biopsies are submitted. The difference of mutations profiles we observe in our study may be a useful additional diagnostic aid in some settings.

A limitation of our study is the lack of detailed clinical data, including therapy and follow-up information. Strengths of our study are the considerable number of vascular tumors included in the analysis (*n* = 104), which represent the heterogenous presentation observed in a routine clinical setting, and the use of a next-generation-sequencing approach screening with high sensitivity simultaneously for many genes known to be relevant in the pathogenesis of vascular proliferations.

In conclusion, we could show that in contrast to malignant vascular proliferations, benign vascular proliferations, in particularly cherry (senile) hemangioma frequently harbor mutually exclusive activating mutations in *GNA14*, *GNA11* and *GNAQ*. *RAS* mutations were less common however, observed in both hemangioma and angiosarcomas. The differences in mutation profile between benign and malignant cutaneous vascular tumors could be applied as a diagnostic aid in distinguishing these entities.

## Data Availability Statement

The datasets presented in this study can be found in online repositories. The names of the repository/repositories and accession number(s) can be found below: Bioproject/Genbank (ID: PRJNA717731).

## Ethics Statement

The studies involving human participants were reviewed and approved by ethics committee of the University of Duisburg-Essen (IRB-number 20–9688-BO). The patients/participants provided their written informed consent to participate in this study.

## Author Contributions

KG and PJ: conceptualization, methodology, visualization and supervision, and project administration. FS, PJ, and KG: software. PJ, DS, EH, and KG: validation. HM, JS, TM, EH, and KG: histological analysis. PJ, IM, and KG: data curation. PJ, HM, TM, AP, EH, and KG: writing—original draft preparation. All authors: writing—review and editing, investigation. All authors have read and agreed to the published version of the manuscript.

## Conflict of Interest

The authors declare that the research was conducted in the absence of any commercial or financial relationships that could be construed as a potential conflict of interest. The reviewer BS declared a past co-authorship with the authors DS, PJ, AP, AS, KG, EH, and AZ to the handling editor.
