# Supplementary material for: GNA14, GNA11, and GNAQ Mutations Are Frequent in Benign but Not Malignant Cutaneous Vascular Tumors
Source: Front Genet. 2021 Apr 30;12:663272. doi: 10.3389/fgene.2021.663272 (PMC8141909; doi:10.3389/fgene.2021.663272)
Supplement: Supplementary file 1 [file Table_1.DOCX]

**SUPPLEMENTAL TABLE**

| **Nr.** | **Gene** | **Chr.** | **Location GRCh37** | **Target exons** | **Selection of relevant mutations covered** | **Primer pairs** |
| --- | --- | --- | --- | --- | --- | --- |
| 1 | *BRAF* | 7 | 140453065 | 11, 15 | G463, G465,V600 | 4 |
| 2 | *NRAS* | 1 | 115256411 | 1, 2 | G12, G13, Q61 | 5 |
| 3 | *HRAS* | 11 | 533850 | 1, 2 | G12, G13, Q61 | 2 |
| 4 | *KRAS* | 12 | 25380250 | 1, 2 | G12, G13, Q61 | 2 |
| 5 | *KIT* | 4 | 455593572 | 11, 13, 17 | L576, K642, N822 | 3 |
| 6 | *GNAQ* | 9 | 80409369 | 4, 5 | R183, Q209 | 6 |
| 7 | *GNA11* | 19 | 3114932 | 4, 5 | R183, Q209 | 3 |
| 8 | *CYSLTR2* | 13 | 49281314 | 1 | L129 | 1 |
| 9 | *PLCB4* | 20 | 9389740 | 20 | D630 | 1 |
| 10 | *SF3B1* | 2 | 198267458 | 14 | R625 | 1 |
| 11 | *EIF1AX* | X | 20156647 | 1, 2 | Mutations in exons 1 and 2 | 3 |
| 12 | *BAP1* | 3 | 5243501 | all (17) | Mutations in all exons | 46 |
| 13 | *SRSF2* | 17 | 74732226 | all (2) | Mutations in all exons | 9 |
| 14 | *GNA14* | 9 | 80043813 | 4, 5 | R179, Q205 | 3 |
| 15 | *GNA15* | 19 | 3151695 | 4, 5 | R186, Q212 | 4 |

**Supplemental Table 1. Genes covered in the applied sequencing panel**
